# Emulsion-Based Intradermal Delivery of Melittin in Rats

**DOI:** 10.3390/molecules22050836

**Published:** 2017-05-19

**Authors:** Sang Mi Han, Se Gun Kim, Sok Cheon Pak

**Affiliations:** 1 Rural Development Administration, National Academy of Agricultural Science, Wanju, Chonbuk 55365, Korea; kimsegun@korea.kr; 2 School of Biomedical Sciences, Charles Sturt University, Bathurst, NSW 2795, Australia; spak@csu.edu.au

**Keywords:** bee venom, emulsion, melittin, permeation

## Abstract

Bee venom (BV) has long been used as a traditional medicine. The aim of the present study was to formulate a BV emulsion with good rheological properties for dermal application and investigate the effect of formulation on the permeation of melittin through dermatomed rat skin. A formulated emulsion containing 1% (*w*/*v*) BV was prepared. The emulsion was compared with distilled water (DW) and 25% (*w*/*v*) *N*-methyl-2-pyrrolidone (NMP) in DW. Permeation of melittin from aqueous solution through the dermatomed murine skin was evaluated using the Franz diffusion cells. Samples of receptor cells withdrawn at pre-determined time intervals were measured for melittin amount. After the permeation study, the same skin was used for melittin extraction. In addition, a known amount of melittin (5 μg/mL) was added to stratum corneum, epidermis, and dermis of the rat skin, and the amount of melittin was measured at pre-determined time points. The measurement of melittin from all samples was done with HPLC-MS/MS. No melittin was detected in the receptor phase at all time points in emulsion, DW, or NMP groups. When the amount of melittin was further analyzed in stratum corneum, epidermis, and dermis from the permeation study, melittin was still not detected. In an additional experiment, the amount of melittin added to all skin matrices was corrected against the amount of melittin recovered. While the total amount of melittin was retained in the stratum corneum, less than 10% of melittin remained in epidermis and dermis within 15 and 30 min, respectively. Skin microporation with BV emulsion facilitates the penetration of melittin across the stratum corneum into epidermis and dermis, where emulsified melittin could have been metabolized by locally-occurring enzymes.

## 1. Introduction

Bee venom (BV) has long been used in East Asian countries as a traditional medicine. The venoms of bees are complex mixtures of biologically-active proteins and peptides, such as phospholipases, hyaluronidase, phosphatase, α-glucosidase, serotonin, histamine, dopamine, noradrenaline, and adrenaline. In addition, melittin, apamin, and mast cell degranulating peptide are also found in BV. A recent review article on bee venoms for their potential therapeutic and biotechnological applications in biomedicine focuses on two major peptides—namely, melittin and apamin [1]. Both melittin and apamin have a broad spectrum of therapeutic applications.

Topical application of cosmetics containing purified BV has been reported to be effective in the treatment of humans with acne vulgaris [2,3]. Another study conducted by An et al. [4] reported that BV has a potential anti-bacterial effect against inflammatory skin disease. In this context, *Propionibacterium acnes* was intradermally injected into the ears of mice. Following the injection, BV mixed with Vaseline was applied to the skin surface of the ear. Histological observation revealed that the *P. acnes* injection induced a considerable increase in the number of infiltrated inflammatory cells and inflammatory cytokines. By contrast, the BV-treated ears showed noticeably reduced ear thickness. Moreover, heat-killed *P. acnes* increased the secretion of cytokines in human keratinocytes and monocytes, which was reversed by BV treatment [5]. Further studies have demonstrated that BV application induced a significant anti-inflammatory response via inhibition of inflammatory mediators, similar to what is achieved by treatment with non-steroidal anti-inflammatory drugs. Han et al. [6] have shown that BV treatment has anti-inflammatory effects in the skin and a rapid cicatrizing effect on wounds in rats. Intraperitoneal administration of BV inhibited the degranulation of mast cells and the production of pro-inflammatory cytokines in compound 48/80-treated mouse skin tissues [7]. BV also inhibited the compound 48/80-induced activation of nuclear factor kappa B (NF-κB), which regulates pro-inflammatory cytokine expression. Lee et al. [8] demonstrated that BV and melittin mediated the anti-inflammatory effect via NF-κB signaling, confirming that activation of the p38 pathway was important in the activation of cytokines during inflammatory reactions. Many topical BV-based skincare cosmetic products are available in the market. For example, BV serum treatment clinically improved facial wrinkles by decreasing total wrinkle area, total wrinkle count, and average wrinkle depth [9]. Furthermore, BV serum was found to be effective in the treatment of mild-to-moderate acne vulgaris [3]. However, topical formulations or products of melittin—the most studied and prevalent substance in BV—are presently not available in the market. Moreover, low permeability of melittin across the stratum corneum due to its high molecular weight as well as strong binding to the lipid bilayers in the skin makes its use as a key ingredient in skin care products difficult. An ideal cosmetic skincare product must pass through the stratum corneum and reach the viable epidermis and dermis. Among various strategies to improve the penetration of melittin across the stratum corneum, an emulsification method was used in the current study.

The overall objective of this study was to formulate a BV-based emulsion and access delivery of melittin through dermatomed murine skin using Franz diffusion cells. Skin was then treated with tape stripping to create skin matrices to extract melittin. Finally, another dermatomed rat skin was separated into stratum corneum, epidermis, and dermis before the melittin was applied to them, and retention of melittin in skin matrices was measured.

## 2. Results

### 2.1. In Vitro Skin Permeation Study

Permeation of melittin from aqueous solution through dermatomed murine skin was evaluated. No melittin was detected in the receptor phase at all time points, even after 36 h in all test groups (Table 1).

### 2.2. Extraction of Melittin from Skin

The amount of melittin was further analyzed in stratum corneum (tapes 0–3) and underlying layers (viable epidermis and dermis) using the tape stripping technique. Melittin was still not detected in all skin matrices of all test groups, while melittin remaining in tapes 0–1 of all test groups was almost negligible (Table 2). This finding, as well as data from skin permeation study, indicated the ability of melittin in the form of emulsion to permeate across the skin—probably due to the chemical interaction of emulsified melittin with the lipid bilayers of the skin. It may also be possible that any small amount of emulsified melittin when permeated could have been metabolized by the degradative action of locally occurring enzymes in the skin or have been below the limit of detection of the HPLC-MS/MS method. An additional melittin recovery study was conducted to confirm this implication.

### 2.3. Extraction Recovery Study

**Figure 1:**
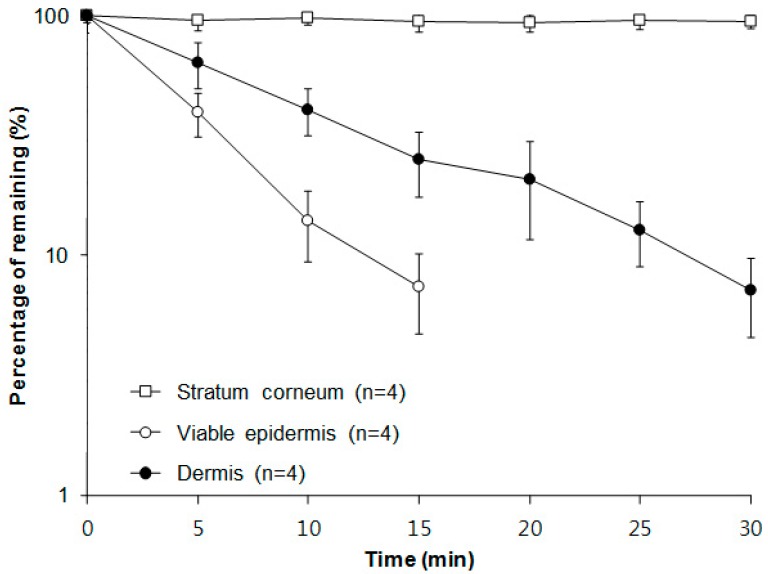
Extraction recovery of melittin from stratum corneum, viable epidermis, and dermis.

Further experiment was carried out using stratum corneum, epidermis, and dermis treated by melittin, because extraction of melittin from dermatomed murine skin following the in vitro skin permeability study was not found to be practically feasible. As shown in Figure 1, the amount of melittin added to all skin matrices was corrected against the amount of melittin recovered. The extraction efficiency of less than 10% for melittin from viable epidermis and dermis was observed in 15 and 30 min, respectively. However, the total amount of melittin retained in the stratum corneum was found to be 100%, confirming the presence of no enzymes for melittin metabolism in the stratum corneum. Although the trend that the amount of melittin gradually reduced from the outer layer to the deeper layers was observed, the metabolizing capacity of melittin was more efficient in epidermis than dermis. In fact, most biotransformation reactions in the rat skin occur predominantly in the epidermis due to both phase I and II reactions [10].

## 3. Discussion

The main purpose of the present study was to evaluate the emulsification effect of BV for dermal application by measuring the amount of melittin remaining from permeation process in the different skin layers. This is the first experimental study to determine the effects of BV-containing emulsion on skin permeation. In this research, we found that emulsified melittin when permeated could have been metabolized by the degradative action of locally-occurring enzymes, at least in the rat skin. 

The mechanism by which BV exerts its therapeutic action on skin disorders has mostly been related to anti-inflammatory activity. When skin bacteria were incubated with BV, the bacteriostatic action of BV was demonstrated which was further supported by its anti-inflammatory activity against skin bacteria through suppression of the secretion of pro-inflammatory cytokines [11]. This in vitro action of antimicrobial property of BV study was further translated into an in vivo study of human subjects with acne vulgaris [2,3] or wrinkles [9]. Repeated exposure to UV irradiation induces an elevated secretion of matrix-degrading enzymes called matrix metalloproteinases (MMPs). With human dermal fibroblasts, BV markedly reduced UV-induced MMP protein levels compared with those of UV-irradiated control [12]. This was attributed to the presence of anti-melanogenic property of BV by inhibiting tyrosinase-related proteins [13]. When wounded mice were treated topically with BV, increased collagen protein synthesis was demonstrated [6] which might be related to increased proliferation and migration of human epidermal keratinocytes [14]. BV as a cosmetic ingredient may be useful as a topical agent for promoting skin regeneration or treatment of certain epidermal conditions. The topical application of BV can be well tolerated in the human skin because it exhibited no dermal irritation potential in animal studies [15].

Despite the widespread use of BV, topical formulations or products of melittin are not available in the market due to its low permeability across the skin layers. This study showed an enhancement in the delivery of melittin from aqueous solution to deeper skin layers of epidermis and dermis. Furthermore, melittin in the form of emulsion was not detected in the receptor phase in the in vitro permeation study using dermatomed rat skin. In conclusion, bee venom emulsion formulated using 1% BV and triethanolamine as a base solubilizing oils and other ingredients was found to be rheologically stable and acceptable for topical application. The permeability of melittin from the solution to the deeper skin layers for an ultimate metabolism was enhanced with the use of emulsification. As epidermis and dermis are the ideal sites for the biological effects of melittin, enhancement in its intradermal delivery mediated by emulsification seems to have a bright future for its use as an anti-photoaging agent in cosmetic products.

## 4. Materials and Methods

### 4.1. Purified Honeybee Venom Collection

Bee venom added to cosmetic emulsion was from the experimental colonies of natural honey bees (*Apis mellifera* L.) that were maintained at the National Institute of Agricultural Science, Korea. Bee venom was collected with a bee venom collector (Chungjin Biotech, Ansan, Korea) in a sterile manner under strict laboratory conditions. In brief, the bee venom collector was placed on the hive, and the bees were given enough electric shock to cause them to sting a glass plate from which dried bee venom was later scraped off. The collected venom was diluted in cold sterile water and then centrifuged at 10,000× *g* for 5 min at 4 °C to discard residues from the supernatant. Purified bee venom was lyophilized by freeze dryer and refrigerated at 4 °C for later use.

### 4.2. Formulation of Emulsion

The basic composition for emulsion formulation includes lanolin (8.5% *w*/*v*), petrolatum (4.2% *w*/*v*), stearic acid (6.8% *w*/*v*), and propyl parahydroxybenzoate (0.05% *w*/*v*). Further ingredients of 1% (*w*/*v*) BV, methyl parahydroxybenzoate (0.1% *w*/*v*), disodium edentate (0.05% *w*/*v*), propylene glycol (5% *w*/*v*), and triethanolamine (1% *w*/*v*) were added for the final formulation (Table 3). In addition to emulsion, distilled water (DW) and 25% (*w*/*v*) *N*-methyl-2-pyrrolidone (NMP) in DW were used for comparison.

### 4.3. Preparation of Skin Samples

The animal protocols for this study were approved by the boards of the Catholic University of Daegu and Daegu Catholic University Medical Center (Daegu, Korea). Male Sprague-Dawley rats (230 ± 20 g, Samtako Bio Korea Co., Ltd. Osan, Korea) were used to obtain the skin sections. The animals were euthanized by high concentration of diethyl ether and the dorsal hair of rats was shaved with electric clipper (Model 808, Daito Electric Co., Osaka, Japan). Full-thickness skin (5 × 5 cm^2^) was prepared by excising the dorsum of rats, cleaned thoroughly using distilled water and stored at −20 °C until further use. It was then dermatomed using a Dermatome to obtain skin pieces, approximately 0.6 mm thick. The dermatomed skin was then cut into approximate sizes (5 × 5 cm^2^) for mounting on the vertical Franz diffusion cells (Bioneer, Hørsholm, Denmark).

### 4.4. In Vitro Skin Permeation Study

Permeation experiment of melittin through dermatomed murine skin was performed with a system employing Franz diffusion cells. The temperature in the receptor phase was maintained at 32 ± 0.5 °C with an external constant temperature circulating water bath. Skin was mounted on a receptor phase (12 mL) with the dermis facing the receptor and stratum corneum towards the donor phase with effective permeation area of 1.766 cm^2^. The receptor and donor phases were filled with phosphate-buffered saline (PBS) solution, and the receptor fluid was continuously stirred with a magnetic bar at 600 rpm to maintain homogeneity. After 1 h equilibration, the solution in the receptor phase was replaced with fresh PBS, and emulsion was applied on the skin in the donor phase. The donor phase was then covered with a parafilm to avoid any evaporation process. The samples of the receptor cell (0.2 mL) were withdrawn at pre-determined time points (1, 2, 3, 4, 5, 6, 8, 10, 12, 24, and 36 h after DW, 25% NMP, and emulsion application) and replaced with equal volume of PBS buffer to keep a constant volume. Samples were analyzed using HPLC-MS/MS (Waters, Minneapolis, MA, USA).

### 4.5. Extraction of Melittin from Skin

After the permeation study, donor emulsion was removed first, and the skin was then cleaned properly with PBS buffer. To measure the amount of melittin retained in the skin layers, stratum corneum was separated from the underlying epidermis and dermis using tape stripping. Adhesive tapes were applied onto the permeation area of the skin, one by one, pressed manually with a finger, removed quickly with forceps, and collected in six-well plates. Tapes 0–3 were collected separately to measure the amount of melittin remaining in stratum corneum. The remaining viable epidermis and dermis were cut into small pieces using surgical scissors. Skin pieces were weighed, and 25 mg of skin was placed in separate vials. The vials were centrifuged at 20,000× *g* for 20 min at room temperature so as to ensure that all the skin pieces were at the bottom of the vial and then kept overnight in an incubator at 37 °C. Ethanol (500 μL) was then added to each vial and vortexed to remove any emulsion present on the skin surface. The skin pieces were then removed and placed individually from each vial in a six-well plate. Methanol (2 mL) was added to each well. The plates were kept on the shaker at 150 rpm for 4 h. Samples were then filtered through 0.22 μm syringe filters and analyzed by HPLC-MS/MS .

### 4.6. Extraction Recovery Study

The protocol as described in the skin sample preparation was followed. Dermatomed murine skin was tape-stripped to separate the stratum corneum from epidermis and dermis. Stratum corneum, epidermis, and dermis were cut into small pieces, and placed in six-well plate. Melittin standard solution (5 μg/mL) was added into respective vials containing the weighed amount of skin. The vials were kept at room temperature to ensure that all the skin pieces were in complete contact with melittin to maximize its absorption into skin layers. The amount of 25 μL of samples were withdrawn at pre-determined time intervals (0, 5, 10, 15, 20, 25, and 30 min; for epidermis, it was up to 15 min), and acetonitrile (100 μL) was added to stop the enzymatic reaction and precipitate the protein in the skin. Each vial was vortexed and centrifuged at 4000× *g* for 10 min. The supernatant was used to analyze the amount of melittin using HPLC-MS/MS.

### 4.7. Quantitative Analysis

HPLC-MS/MS was used for the quantitative estimation of melittin. A Waters 2690 HPLC system (Waters, Minneapolis, MA, USA) coupled with a binary pump, an autosampler, and triple quardruple mass spectrometer equipped with a turbo electrospray ionization source was used. The separation was carried out using Halo C18 column (2.1 × 50 mm, 2.7 μm) from Advanced materials technology (Wilmington, DE, USA). The chromatographic conditions included a flow rate of 0.3 mL/min, injection volume of 5 μL, column temperature of 30 °C, solvent A of MeCN containing 0.1% formic acid, solvent B of H_2_O containing 0.1% formic acid, and a gradient elution of 95% for 1 min, 95–30% B in 3.5 min, 30–95% B in 10 min. Mass spectrometric analysis was conducted in positive ion mode under the following settings: gas temperature 320 °C, curtain gas 4 psi, collision gas 25 psi, DP 31 V, FP 370 V, EP 3.5 V, CE 61 V and CXP 2 V. Quantification of melittin was estimated by scanning the following multiple-reaction monitoring transition: melittin (*m*/*z* 570.1→86.2, dwell time 300 ms).

## 5. Conclusions

Skin microporaton with BV emulsion facilitates the skin permeability of melittin which may be due to enzyme activity.

## Figures and Tables

**Table 1:** Concentrations of melittin in receptor phase.

Time (h)	DW ^a^	25% NMP ^b^	Emulsion
1	ND ^c^	ND	ND
2	ND	ND	ND
3	ND	ND	ND
4	ND	ND	ND
5	ND	ND	ND
6	ND	ND	ND
8	ND	ND	ND
10	ND	ND	ND
12	ND	ND	ND
24	ND	ND	ND
36	ND	ND	ND

^a^ DW: Distilled water. ^b^ 25% NMP: 25% (*w*/*v*) *N*-methyl-2-pyrrolidone in DW. ^c^ ND: not detected.

**Table 2:** Concentrations of melittin in various matrices of skin.

Matrices	DW ^a^	25% NMP ^b^	Emulsion
Tape 0	<0.05%	<0.05%	<0.05%
Tape 1	<0.05%	<0.05%	<0.05%
Tape 2	ND ^c^	ND	ND
Tape 3	ND	ND	ND
Viable epidermis	ND	ND	ND
Dermis	ND	ND	ND

^a^ DW: Distilled water. ^b^ 25% NMP: 25% (*w*/*v*) *N*-methyl-2-pyrrolidone in DW. ^c^ ND: not detected.

**Table 3:** Composition of emulsion containing 1% bee venom.

	Ingredients	% *w*/*v*
Phase 1	Lanolin	8.5
	Petrolatum	4.2
	Stearic acid	6.8
	Propyl parahydroxybenzoate	0.05
Phase 2	Bee venom	1
	Methyl parahydroxybenzoate	0.1
	Disodium edentate	0.05
	Propylene glycol	5
	Triethanolamine	1
	Purified water	73.3
